# Textual Inference Comprehension in Mild Cognitive Impairment: The Influence of Semantic Processing and Verbal Episodic Memory

**DOI:** 10.3389/fnagi.2021.735633

**Published:** 2021-10-04

**Authors:** Maria Paula Maziero, Ariella Fornachari Ribeiro Belan, Marina von Zuben de Arruda Camargo, Marcela Lima Silagi, Orestes Vicente Forlenza, Marcia Radanovic

**Affiliations:** ^1^Department of Psychiatry, School of Medicine, University of São Paulo, São Paulo, Brazil; ^2^Department of Speech, Language and Hearing Sciences, Federal University of São Paulo, São Paulo, Brazil

**Keywords:** mild cognitive impairment (MCI), inference, comprehension, text, semantic processing, verbal episodic memory

## Abstract

Language complaints, especially in complex tasks, may occur in mild cognitive impairment (MCI). Various language measures have been studied as cognitive predictors of MCI conversion to Alzheimer's type dementia. Understanding textual inferences is considered a high-demanding task that recruits multiple cognitive functions and, therefore, could be sensitive to detect decline in the early stages of MCI. Thus, we aimed to compare the performance of subjects with MCI to healthy elderly in a textual inference comprehension task and to determine the best predictors of performance in this ability considering one verbal episodic memory and two semantic tasks. We studied 99 individuals divided into three groups: (1) 23 individuals with amnestic mild cognitive impairment (aMCI), (2) 42 individuals with non-amnestic mild cognitive impairment (naMCI), (3), and (4) 34 cognitively healthy individuals for the control group (CG). A reduced version of The Implicit Management Test was used to assess different types of inferential reasoning in text reading. MCI patients performed poorer than healthy elderly, and there were no differences between MCI subgroups (amnestic and non-amnestic). The best predictors for inference-making were verbal memory in the aMCI and semantic tasks in the naMCI group. The results confirmed that the failure to understand textual inferences can be present in MCI and showed that different cognitive skills like semantic knowledge and verbal episodic memory are necessary for inference-making.

## Introduction

The concept of mild cognitive impairment (MCI) refers to an intermediate condition between normal cognition and early dementia, where individuals present some degree of cognitive impairment but maintain the preservation of functionality, progressing to full-blown dementia at a rate of 10–15% per year (Albert et al., 2011). MCI is classified as amnestic (a-MCI) and non-amnestic (naMCI) underpinned on memory damage (Kelley and Petersen, 2007). Impairment in episodic memory appears early in MCI patients who will develop Alzheimer's disease (AD) (McKhann et al., 2011).

Many pieces of research have been conducted for the early detection of cognitive decline in MCI (Vega and Newhouse, 2014; Chehrehnegar et al., 2019). Early detection provides better opportunities for pharmacological and non-pharmacological treatments and, therefore, may delay the evolution of the disease (Vega and Newhouse, 2014; Eshkoor et al., 2015; Zetterberg and Bendlin, 2021). Biomarkers of cerebral amyloid and tau deposition through cerebrospinal or neuroimaging studies are expensive, invasive, and unsuitable at the primary care level, especially in developing countries. Thus, the search for cognitive markers indicating the progression from MCI to dementia is of paramount importance (Briceño et al., 2020; Silva et al., 2020).

Compared to other cognitive domains, the linguistic decline in MCI is less studied, but various language measures are being identified as predictors of MCI conversion to AD (Belleville et al., 2017). Reports of language deficits include failures in several tasks such as, verbal fluency, confrontation naming, word definition, sentence comprehension, and repetition, and discourse production (Mueller et al., 2018; McCullough et al., 2019; de la Hoz et al., 2021). Non-literal language deficits as comprehension of proverbs, idiomatic expressions, and non-literal text were also identified in individuals with MCI (Cardoso et al., 2014). In this direction, studies show that the ability to understand inferences may also be affected in MCI (Schmitter-Edgecombe and Creamer, 2010; Gaudreau et al., 2015; Silagi et al., 2021).

Inferential processing is the ability to build mental representations for the complete comprehension of information that is heard or read, based on the application of personal knowledge added to the explicit information expressed, establishing associations and relations, allowing the comprehension of implicit information (Gutiérrez-Calvo, 1999).

Verbal and written communication requires different types of inferential reasoning. The continuous realization of inferences is critical to discourse comprehension since not all information is explicitly conveyed, and some degree of “predictions” and “deductions” about what the speaker or writer “really” means is often necessary to maximize communication effectiveness. The comprehension of inferences is based on well-developed semantic integration and verbal memory skills (Van Dijk and Kintsch, 1983; McNamara et al., 2007).

Thus, the ability to understand textual inferences is considered a high-demanding task that recruits multiple cognitive functions and, therefore, could be sensitive to detect cognitive decline in the early stages of MCI. Furthermore, considering the importance of early detection of decline in cognitive skills in a population facing increasingly more extended life expectancy and the pivotal role of inference comprehension in maintaining effective communication, we aimed to study the inferential comprehension from reading on a cohort of MCI patients. Therefore, we aimed to compare the performance of subjects with MCI to healthy elderly in a textual inference comprehension task and to determine the best predictors of performance in this ability considering one verbal episodic memory and two semantic tasks.

## Materials and Methods

### Participants

We studied 99 elderly followed up at a Psychogeriatric outpatient clinic linked to a university hospital, aged between 60 and 90 years, who presented cognitive complaints or previous diagnosis of cognitive deficits, and a group of elderly engaged as volunteers in studies concerning cognition.

Individuals enrolled in the study were classified into three groups, paired according to age and educational level:

1. CG (*n* = 34): control group with cognitively healthy individuals;
2. aMCI (*n* = 23): individuals with amnestic mild cognitive impairment;
3. naMCI (*n* = 42): individuals with non-amnestic mild cognitive impairment;

First, all participants were evaluated by the Montreal Cognitive Assessment (MoCA) (Nasreddine et al., 2005; Memória et al., 2013) for general cognitive screening, the Geriatric Depression Scale-15 (GDS-15) (Sheikh and Yesavage, 1986; Almeida and Almeida, 1999) to detect depressive symptoms, and the Lawton and Brody Scale for Daily Life Activities (L and B) (Lawton and Brody, 1969; Santos and Júnior, 2012) to assess functional independence for instrumental activities of daily living. Subsequently, the participants were submitted to a neuropsychological assessment, composed of the following tests: Rivermead Behavioral Memory Test (RBMT) (Wilson et al., 1989; Yassuda et al., 2010); Trail Making Test (TMT) (Spreen and Strauss, 1998; Campanholo et al., 2014); Digit Span (DS) (Wechsler, 1997; Nascimento, 2004), FAS-COWA (Spreen and Strauss, 1998); Rey Auditory Verbal Learning Test (RAVLT) (Rey, 1964; Malloy-Diniz et al., 2007), and Rey-Osterrieth Complex Figure (ROCF) (Osterrieth, 1944; Oliveira et al., 2004).

MCI patients were selected based on the criteria of Albert et al. (2011), which cover: (1) cognitive complaint, preferably confirmed by a relative or close person; (2) objective cognitive impairment in one or more cognitive domains, with performance below the expected for peers in the same age range and educational level; (3) not being demented, with preserved simple activities of daily living.

This group was further subdivided into aMCI (evidence of episodic memory impairment) and naMCI (evidence of impairment in cognitive functions other than episodic memory). The inclusion criteria for the MCI subgroups were performance at least 1.5 SD below the mean score on one function and/or between 1 and 1.5 SD below the mean score in two neuropsychological tests of the same function (Petersen, 2004).

All patients diagnosed as MCI had their blood counts, biochemical and lipid profile, vitamin B12, folic acid, syphilis serology, and thyroid function tested and performed magnetic resonance imaging studies to rule out metabolic, infectious, and vascular etiologies for cognitive decline.

Participants with no previous complaints of cognitive decline and who performed normally on cognitive tests were allocated to the GC.

An experienced multidisciplinary team that included neurologists, geriatricians, neuropsychologists, and speech-language pathologists gave the final diagnosis for the groups.

The exclusion criteria for all groups were: being illiterate; having a health condition that could preclude the realization of neuropsychological tests (such as, non-correctable vision or hearing impairment); history or evidence of cerebrovascular injury, non-AD dementias, and other neurologic/psychiatric conditions that might impair cognition (such as, severe traumatic brain injury, epilepsy, depression, bipolar disorder, and psychosis).

The local Ethics Committee approved the study under protocol number CEP 3.318.162, and all participants or their proxies signed a consent form before enrollment in the study.

### Instruments

After cognitive evaluation, the individuals were submitted to a protocol designed to assess inferential, semantic, and verbal episodic memory abilities.

Textual inference comprehension was assessed using The Implicit Management Test (IMT) (in the original French version, *La Gestion de l'Implicite*) (Duchêne May-Carle, 2000), adapted to Brazilian Portuguese (Silagi et al., 2014). The test evaluates the ability to comprehend inferences during reading activities and is designed to assess adult subjects with cognitive and/or communication complaints. The test was applied in a reduced-version containing ten texts consisting of short stories involving two people or describing a verbal interaction; individuals must read and answer three questions for each text. The only admissible answers are: “Yes”, “No”, or “I cannot answer.” The texts contain explicit and implicit information, which is necessary for the correct interpretation during reading. The texts were available for the patients to consult while they answered the questions.

Questions regarding the texts are subdivided into five categories that require different types of inferential reasoning: (1) explicit–questions are answered by using information supplied in the text; no inference-making needed; (2) logical–questions are answered by using a cause-effect relationship with the information provided in the text, through formal reasoning; (3) distractor–questions that have “I cannot answer” as the only possible correct answer, as the information required for an appropriate answer cannot be extracted from the text either explicitly or implicitly; (4) pragmatic–questions are answered by using context and previous experience; and (5) “other”–questions require both logical and pragmatic reasoning. Examples of the different types of questions can be seen in Table 1.

**Table 1:** Types of questions in the implicit management test.

**Type of question**	**Example**
*Explicit*	Nadia called Lucas and told him: “My goodness, have you seen the time?”, and Lucas answered: “Yes, I know, but I cannot find my car keys.” *Has Lucas lost the keys to his car?*
*Logical*	My neighbor's cat never meows, except when it has not eaten for a long time. Today, I heard the cat meowing all morning. *Did my neighbor feed her cat this morning?*
*Pragmatic*	After the weather report, Brigitte said to herself: “I must not forget my umbrella tomorrow.” *Does Brigitte like getting wet?*
*Other*	Peter says: “It costs a lot of money to go to Canada; I cannot go there right now.” *Does Peter have much money right now?*
*Distractor*	Rose says to Suzanne: “Stop eating or you will put on weight!” and Suzanne replies: “So what, men like it.” *Is Rose married?*

Semantic tests included: (a) the Word-Picture Matching (WPM) Test (Weintraub, 2000). This test evaluates spoken word recognition and assesses the frequency of semantic errors in word comprehension. The stimuli consist of five displays, each one containing pictures of four objects that are semantically related. Each display is presented four times (once for each picture as the target) in a total of 20 trials. The presentation order of displays is pseudo-randomized so that no four-picture display appears in sequential trials. One point is given for each correct response given at the first attempt, which allows a maximum score of 20—“I do not know” is considered as an incorrect response; (b) the Semantic Associates Test (SA) (Weintraub, 2000). This test comprises16 displays containing three items presented as two pairs (one target and one distractor), which evaluates the functional, contextual and categorical relationship between the items. The individual is asked to look at the two pairs of pictures in each display (semantically related or non-related) and point to the matching set. For example, in a display containing the pairs: *sweater-blanket/sweater-pillow*, the correct answer is *sweater-blanket* (functional relationship); for the two pairs *sweater-chest/sweater-workbench*, the correct answer is *sweater-chest* (contextual relationship). One point is given for each correct match choice, which allows a maximum score of 16.

To assess verbal and non-verbal episodic memory, we used the Three-Words Three Shapes Test (3W3S) (Weintraub, 2000). The participants are asked to copy three words (*pride, hunger, station*) and three geometric shapes displayed on a sheet of paper and then reproduce them from memory to assess immediate and delayed recall (after five minutes, during which the individuals performed a task of picture description). The test provides scores for verbal memory (immediate and delayed recall for words; WImR and WDR, respectively) and non-verbal memory (immediate and delayed recall for shapes). We considered only the verbal memory scores in this study: each word correctly retrieved in each condition is scored as five, giving a total score of 15. The score of five for each correct word allows for deductions due to adding, subtracting, or substitution errors in writing.

### Statistical Analysis

Intergroup comparison of means for demographic and clinical continuous variables was performed through one-way analysis of variance with Bonferroni's post-test. The Chi-Squared test was used to assess intergroup differences in sex distribution. Mixed-effects linear regression models were used to (a) determine the best predictors for IMT total scores and scores in each question subtype in the total sample, using such scores as dependent variables, schooling and scores on WPM, SA, subtests of the 3W3S as fixed-factors, and within-subjects differences in performance as a random effect; (b) determine the best predictor for IMT in each diagnostic subgroup using diagnosis as the dependent variable, schooling, and scores on WPM, SA, subtests of the 3W3S as fixed-factors, and within-subjects differences in performance as a random effect. Schooling was tested in interaction with all variables. The Akaike information criterion (AIC) was employed to compare the models and choose the best among them, which were those with the smaller AIC values. Model estimations were performed using restricted maximum likelihood (REML). Significance levels were set at *p* < 0.05 values. We used the SPSS^®^ Statistics software version 25 for all analyses.

## Results

The demographic and clinical characteristics of the sample are shown in Table 2. There were no differences between groups on age and sex. The complete neuropsychological evaluation is displayed in the Appendix.

**Table 2:** Demographic variables and performance on tests according to diagnostic group.

**Variable**	**controls (*****n*** **= 34)**	**aMCI(*****n*** **= 23)**	**naMCI (*****n*** **= 42)**	***p*** **-value**	**Multiple comparisons ***p*** < 0.05**
Age (years)	70.8 (7.8)	70.6 (6.7)	69.7 (6.8)	0.768	NA
Schooling (years)	14.4 (3.0)	11.6 (4.7)	11.6 (4.7)	0.088	NA
Sex *F*	20	11	20	0.749	NA
*M*	14	12	22		
IMT	25.1 (2.4)	20.0 (7.3)	22.6 (4.2)	<0.001	CG ≠ aMCI, naMCI
*Pragmatic*	7.8 (1.2)	7.2 (1.9)	7.8 (1.4)	0.533	NA
*Distractor*	6.4 (0.8)	5.5 (1.7)	5.3 (2.2)	0.072	NA
*Explicit*	3.7 (0.4)	3.3 (0.9)	3.4 (0.8)	0.448	NA
*Logical*	4.1 (0.8)	3.3 (1.2)	3.5 (1.3)	0.044	CG ≠ aMCI
*Other*	3.3 (1.2)	2.8 (1.4)	2.6 (1.2)	0.115	NA
WPM	19.9 (0.1)	19.9 (0.0)	19.9 (0.2)	0.154	NA
SA	15.8 (0.5)	15.9 (0.8)	15.5 (1.5)	0.628	NA
3W3S					
*WImR*	13.9 (2.4)	12.3 (3.7)	10.3 (4.9)	0.004	CG ≠ naMCI
*WDR*	14.4 (1.9)	11.8 (5.5)	12.5 (3.6)	0.016	CG ≠ aMCI, naMCI

*Data displayed as Mean (SD) except for sex (number of individuals); aMCI, amnestic mild cognitive impairment; naMCI, non-amnestic mild cognitive impairment; F, female; M, male; IMT, Implicit Management Test; WPM, Word-Picture Matching; SA, Semantic Associates; 3W3S, Three Words Three Shapes; WImR, Words Immediate Recall; WDR, Words Delayed Recall; NA, not applicable*.

The performance of the sample in the IMT, WPM, SA, and 3W3S tests is shown in Table 2. MCI patients performed worse than controls in the IMT total score; aMCI patients performed worse than controls in “logical” questions, though in the margin of statistical non-significance. In the 3W3S test, MCI patients performed worse than controls in WImR. There were no intergroup differences in the WPM and SA tests; aMCI and naMCI patients performed similarly in all tests.

Mixed-effect regression models showed that the best predictors for total IMT performance for the whole sample were schooling and the verbal episodic memory tasks; however, predictors changed according to the type of question: SA for pragmatic questions, schooling, SA and WDR for distractor questions, WPM for explicit questions, and schooling for logical questions. There were no predictors for “other” questions (Table 3). We also found different main predictors for inference-making performance across diagnostic groups. In the control group, schooling and WPM were the best predictors, with a trend for SA. The best predictor was verbal memory in the aMCI group, while in the naMCI group, inference-making skills were associated with semantic tasks (WPM and SA) (Table 4).

**Table 3:** Mixed-effect linear regression for predictors of IMT scores according to the type of question in the whole sample.

***Type of question*** **/predictor**	**Estimated coefficient**	**SE**	***F***	***t***	***p-value***	**CI 95%**	
						**Lower bound**	**Upper bound**
*Total*							
Schooling	0.35	0.10	10.95	3.31	0.001	0.14	0.57
WImR	0.28	0.10	7.26	2.69	0.009	0.07	0.49
WDR	0.31	0.12	6.82	2.61	0.011	0.07	0.56
*Pragmatic*							
SA	0.46	0.14	10.43	3.23	0.02	0.17	0.74
*Distractor*							
Schooling	0.15	0.40	14.43	3.79	<0.001	0.07	0.23
SA	0.39	0.14	7.28	2.70	0.008	0.10	0.68
WDR	0.11	0.04	7.44	2.72	0.008	0.03	0.20
*Explicit*							
WPM	1.29	0.39	10.81	3.28	0.001	0.50	2.07
*Logical*							
Schooling	0.06	0.02	5.60	2.36	0.02	0.01	0.12
WDR	0.08	0.03	6.68	2.58	0.01	0.02	0.15

*Dependent Variable, Implicit Management Test (IMT); Standard Error; CI, Confidence Interval; WImR, Words Immediate Recall; WDR, Words Delayed Recall; SA, Semantic Associates; WPM, Word-Picture Matching+*.

**Table 4:** Mixed-effect linear regression for predictors of IMT scores by diagnostic group.

**Group predictors**	**Estimated coefficient**	**SE**	***F***	***t***	***p-value***	**CI 95%**	
						**Lower bound**	**Upper bound**
*CG*							
Schooling	0.44	0.14	9.76	3.12	0.00	0.15	0.74
WPM	−8.54	2.91	8.59	−2.93	0.00	−14.52	−2.55
SA	1.80	0.88	4.19	2.04	0.05	0.00	3.61
*aMCI*							
WImR	1.13	0.38	8.81	2.96	0.01	0.31	1.94
*naMCI*							
WPM	5.50	2.27	5.85	2.41	0.02	0.89	10.11
SA	1.19	0.38	9.69	3.31	0.00	0.41	1.97

*Dependent Variable, Implicit Management Test (IMT); aMCI, amnestic mild cognitive impairment, naMCI, non-amnestic mild cognitive impairment; SE, Standard Error; CI, Confidence Interval; WPM, Word-Picture Matching; SA, Semantic Associates; WImR, Word Immediate Recall*.

## Discussion

Inferential processing is a poorly explored ability in MCI. It is considered a complex linguistic skill, mainly in the context of text comprehension, as it depends on both linguistic and domain-general cognitive abilities. As it is a demanding skill, we hypothesized that the ability to understand inferences might already be impaired in patients with MCI and that possible changes in basic language processing and other cognitive functions could interfere with this ability. Thus, the aim of this study was to compare the performance of patients with MCI to a sample of healthy elderly in a textual reading task that requires the understanding of different types of inferences, as well as verifying whether semantic knowledge and episodic verbal memory would be predictors of this ability.

### Performance on the Inference Comprehension, Semantic, and Episodic Verbal Memory Tasks

Regarding the performance of the groups in the inference comprehension test (IMT), we verified that the MCI group showed worse performance than controls in the total score. The aMCI group performed poorer than controls in “logical” questions but with a *p*-value approaching the limit of statistic non-significance. The aMCI and naMCI groups performed similarly regarding total scores and subtypes of questions.

Few studies in the literature have addressed the performance of individuals with MCI in inference comprehension tasks. We found three studies that showed similar results about the difficulty of understanding inferences in subjects with MCI. Schmitter-Edgecombe and Creamer (2010) verified that aMCI subjects produced fewer inferences in a story comprehension task than controls and had more difficulties explaining story events and using preliminary text information to support inference generation. Similarly, Gaudreau et al. (2015) found that MCI participants were impaired in identifying ironic or sincere stories that required mental inference capacities, compared to control subjects. We found only one study that compared aMCI and naMCI on the ability to understand inferences. Silagi et al. (2021) evaluated a different cohort of MCI individuals with the same test used in our study (IMT). They found that MCI patients had difficulty understanding inferences compared to controls and the accuracy analyses showed that the total score in the IMT provided good sensitivity and specificity in discriminating MCI from normal individuals. However, they were also unable to differentiate the MCI subgroups from each other in the task.

As for the semantics tasks, all groups showed similar performance in the WPM and SA tests. We observed a ceiling-effect regarding performance in the WPM task in all groups. Semantic impairment has been widely reported in MCI in tasks ranging from verbal fluency, naming, and sentence comprehension (Emery, 2000; Balthazar et al., 2008; Rinehardt et al., 2014; Silagi et al., 2015). However, studies show that the performance on isolated word comprehension and semantic association (as required in WPM and SA) are usually preserved in the patients until the early stages of AD, that is, the semantic impairments in MCI are associated with difficulty in lexical or lexico-phonological search, and in complex tasks involving a more fine-grained semantic decision, with basic semantic knowledge preserved (Ortiz and Bertolucci, 2005; Barbeau et al., 2012; Kirchberg et al., 2012; Tsantali et al., 2013; Venneri et al., 2018).

On the other hand, the verbal episodic memory tests (3W3S) were able to differentiate between controls and MCI as well established in the literature (Ding et al., 2019; Wasserman et al., 2019; Abraham et al., 2020; Silva et al., 2020).

### Predictors of the Inference Comprehension Ability

Our mixed-effect model showed that schooling and verbal episodic memory tasks were the best predictors for inference-making performance in the whole sample. Regarding the predictors of the ability to understand inferences in different diagnostic groups, we found distinct profiles in healthy controls and MCI groups.

In the control group, schooling and WPM were the best predictors, with a trend for SA. A study with healthy subjects showed a strong effect of education on inference comprehension; individuals with higher educational levels had better performance than individuals with a lower educational level on the total score and across all question types of the inference test (Silagi et al., 2014). The authors discussed that schooling plays an essential role in developing several cognitive-linguistic skills, which support inferential reasoning (Ardila, 2005).

The influence of semantic knowledge was also proven in the ability to understand texts with inferences in healthy subjects (Yeari and van den Broek, 2015, 2016; Dong et al., 2020). Semantic integration is essential for inference making, and it occurs as a result of the activation of the semantic network that produces access to a word's meaning with consequent co-activation of words and meanings of the same semantic category. Inference-making is based on the existence of strong semantic associations with specific ideas conveyed by speech or text. Yeari and van den Broek (2015) found that robust associations between textual information and personal background knowledge result in a greater probability of the information being activated. This activation of inference from background knowledge is a function of the number and strength of semantic constraints imposed by the evoking text.

In the aMCI group, the best predictor of inference comprehension was verbal memory. The influence of episodic memory on the ability to understand inferences is well established and addressed by previous studies in subjects with MCI. Schmitter-Edgecombe and Creamer (2010) reported impairment in the production of inferences in MCI patients associated with poorer delayed verbal memory abilities. The authors argue that episodic memory influences inference comprehension because it helps create causal connections between different text parts and establish an integrated story. Similarly, Gaudreau et al. (2015) showed that the poorer comprehension of mental state inferences in the MCI population was correlated with episodic memory and executive functions difficulties. Finally, Silagi et al. (2021) found correlations between the scores in the inference comprehension task and RAVLT in an aMCI group, presupposing that the impairment in inference comprehension observed in this group was related to episodic memory failure. In contrast, they found correlations between the total test score and the TMT-A in the naMCI group, associating the poor inference comprehension in this group with failure in attention and executive functions (which we did not include in our mixed-effect models).

In our results, in the naMCI group, inference-making skills were associated with the semantic tasks (WPM and SA). According to Graesser et al. (1994), some types of inferences are built when long-term memory knowledge structures are activated and incorporated into the representation of the text's meaning. Van Dijk and Kintsch, 1983 establishes the relationship between inferential processing and how information is stored, highlighting that long-term memory is organized in semantic bundles, hierarchically ordered. Lastly, Rumelhart (1980) defines schema as the data structure for representing concepts stored in memory. Schemas represent knowledge about concepts, events, and actions. According to the author, the schema underlies a concept stored in memory, which generates meaning. Schemas allow access to information stored in memory while reading and building inferences.

As for the different types of IMT questions, we hypothesize that the main predictors reflect the nature of reasoning necessary to successfully identify and extract the information in order to answer the questions related to the texts:

(a) pragmatic inferences, which are based primarily on “world knowledge” (the non-linguistic information that helps a reader or listener interpret the meanings of words and sentences) were associated with SA. This task requires the evaluation of functional, contextual, or semantic-category relations between two words (presented as pictures), thus, assessing the integrity of the semantic network.
(b) distractor inferences were associated with schooling, SA, and verbal memory. This type of inference requires that the individual move from the interpretative approach to acknowledge that the only possible answer is “I cannot answer” since the information required for an appropriate answer cannot be extracted from the text either explicitly or implicitly.
(c) explicit inferences were associated primarily with verbal memory. These questions are answered using information supplied in the text; no inference-making is needed.
(d) logical inferences were associated with educational level. These questions are answered by using a cause-effect relationship with the text's information, employing formal reasoning.

This study belongs to a series in which we explore high-demanding language abilities, including inferential processing (both textual and visual) in the normal aging-AD continuum and stroke patients (Silagi et al., 2014, 2018, 2021). Based on previous results in normal individuals and MCI patients, we traced some variables influencing performance in the IMT, including executive functions and episodic memory (Silagi et al., 2014, 2021). We then decided to proceed with our investigation focusing on the role of semantic abilities in inference-making by employing the WPM and SA tasks. An interesting finding in our study was that by conducting a complementary analysis of the “justifications” (that is, how the stimulus word “matched” the individual's response) in the SA task, we observed that many patients could not explain it or provided nonsense explanations, despite making the correct choice (data not shown). We believe that these observations point to a subtle impairment of semantic processing, albeit not sufficient to prevent a correct choice. We intend to explore these findings in subsequent studies.

This study has some limitations: its cross-sectional design and the need for a larger sample to include a higher number of variables in our predictive model. The latter was precluded by the Covid pandemic that impeded us from proceeding with the enrollment of patients. Another limitation is that, given the ceiling-effect observed in WPM, we could have obtained better results regarding the impact of this semantic ability on inference-making should we have used a more demanding task. More studies, preferably longitudinal, are needed to confirm the results.

## Final Comments

Individuals with MCI have difficulties in understanding inferences during reading. It was possible to differentiate MCI patients from cognitively healthy individuals, but it was not possible to differentiate aMCI from naMCI. Despite this, different predictors seem to influence the performance of groups in this skill. The best predictors for inference-making were verbal memory in the aMCI and semantic tasks in the naMCI group. The results confirmed that failure to perform textual inferences may be present in MCI and showed that different cognitive skills like semantic knowledge and verbal episodic memory are necessary for inference-making. It is also essential to understand the interaction among several basic cognitive abilities that, together, allow for the accomplishment of high-demanding cognitive tasks, such as, inference-making, in order to guide rehabilitation efforts according to the specificities of each patient's deficits.

## Data Availability Statement

The original contributions presented in the study are included in the article/supplementary material, further inquiries can be directed to the corresponding author.

## Ethics Statement

The studies involving human participants were reviewed and approved by Ethics Committee for the Analysis of Research Projects (CAPPesq) from Hospital das Clínicas, School of Medicine, University of São Paulo. The patients/participants provided their written informed consent to participate in this study.

## Author Contributions

All authors contributed to the study conception and design. MM: collected the language data and wrote part of the paper. AB: assisted with collection of language data. MC: collected neuropsychological data. MR: responsible for clinical evaluation, diagnosis, and carried out the statistical analysis. OF: supervised the data collection. MS: wrote the first draft of the manuscript. All authors commented on previous versions of the manuscript. All authors read and approved the final manuscript.

## Funding

The Laboratory of Neuroscience (LIM-27), University of São Paulo, receives financial support from the Alzira Denise Hertzog Silva Association (ABADHS), Fundação de Amparo à Pesquisa do Estado de São Paulo (FAPESP; number 2016/01302-9), and Conselho Nacional de Desenvolvimento Científico e Tecnológico (CNPq; number 466625/2014-6).

## Conflict of Interest

The authors declare that the research was conducted in the absence of any commercial or financial relationships that could be construed as a potential conflict of interest.

## Publisher's Note

All claims expressed in this article are solely those of the authors and do not necessarily represent those of their affiliated organizations, or those of the publisher, the editors and the reviewers. Any product that may be evaluated in this article, or claim that may be made by its manufacturer, is not guaranteed or endorsed by the publisher.

## Appendix

**Table A1:** Neuropsychological assessment of the sample.

**Test**	**Control (*****n*** **= 34)**	**aMCI(*****n*** **= 23)**	**naMCI (*****n*** **= 42)**	***p*** **-values**	**Multiple comparison (***p*** < 0.05)**
***MoCA***	27.6 (1.7)	23.1 (4.0)	24.4 (2.7)	<0.0001*	CG ≠ tMCI
***GDS***	3.8 (4.7)	10.1 (6.8)	7.8 (5.8)	<0.0001*	CG ≠ tMCI
***L and B***	26.6 (1.4)	25.7 (2.1)	26.1 (2.5)	0.370	NA
***RBMT***					
*Screening*	10.2 (1.7)	8.0 (2.6)	9.4 (1.9)	0.001*	CG ≠ aMCI
*Profile*	21.7 (2.4)	17.3 (4.4)	19.8 (3.1)	< 0.0001*	CG ≠ tMCI
***TMT–A***					
*Errors*	0 (0)	0.1 (0.5)	0.1 (0.4)	0.119	NA
*Time*	42.4 (13.2)	62.4 (35.7)	58.4 (25.6)	<0.0001*	CG ≠ tMCI
***TMT–B***					
*Errors*	0.4 (0.7)	1.2 (1.7)	1.1 (1.3)	0.119	NA
*Time*	87.7 (31.9)	143.3 (95.3)	138.0 (91.4)	0.005*	CG ≠ tMCI
***Stroop***					
*Errors*	1.3 (2.6)	2.2 (2.8)	1.6 (2.1)	0.567	NA
*Time*	29.2 (8.6)	40.1 (14.0)	37.3 (13.0)	0.005*	CG ≠ tMCI
***DS***					
*Forward*	9.0 (2.2)	8.3 (2.0)	7.6 (2.0)	0.059	NA
*Backward*	6.7 (1.4)	4.8 (1.8)	4.7 (1.9)	<0.0001*	CG ≠ tMCI
***FAS-COWA***	42.1 (8.5)	35.2 (11.1)	35.5 (11.5)	0.015*	CG ≠ tMCI
***RAVLT***					
*Recall*	10.2 (2.1)	4.5 (2.7)	7.9 (2.6)	<0.0001*	CG ≠ tMCI; aMCI ≠ naMCI
*Total*	47.9 (6.7)	33.2 (7.7)	38.5 (7.8)	<0.0001*	CG ≠ tMCI
***ROCF***					
*Copy*	41.0 (36.0)	36.5 (20.6)	31.9 (5.4)	0.001*	CG ≠ tMCI
*Recall*	20.7 (15.3)	17.86 (18.6)	13.4 (5.7)	0.001*	CG ≠ tMCI

*Data presented as mean (SD); aMCI, amnestic mild cognitive impairment; naMCI, non-amnestic mild cognitive impairment; tMCI, total mild cognitive impairment (aMCI + naMCI); MoCA, Montreal Cognitive Assessment; GDS, Geriatric Depression Scale; L and B, Lawton and Brody Scale for Daily Life Activities; RBMT, Rivermead Behavioral Memory Test; TMT, Trail Making Test; DS, Digit Span; FAS-COWA, Controlled oral word association**–**letters F, A, S; RAVLT, Rey Auditory Verbal Learning Test; ROCF, Rey-Osterrieth Complex Figure; NA, not applicable*.
